# The Munich Shoulder Questionnaire (MSQ): development and validation of an effective patient-reported tool for outcome measurement and patient safety in shoulder surgery

**DOI:** 10.1186/1754-9493-6-9

**Published:** 2012-05-18

**Authors:** Florian Schmidutz, Marc Beirer, Volker Braunstein, Viktoria Bogner, Ernst Wiedemann, Peter Biberthaler

**Affiliations:** 1 Department of Orthopedic Surgery, University of Munich (LMU), Marchioninistrasse 15, 81377, Munich, Germany; 2 Department of Trauma Surgery, Technical University of Munich (TUM), Ismaningerstrasse 22, 81675, Munich, Germany; 3 Department of Shoulder Surgery, University of Munich (LMU), Nussbaumstrasse 20, 80336, Munich, Germany; 4 OCM Munich, Steinerstrasse 6, 81369, Munich, Germany

**Keywords:** Shoulder outcome, Shoulder function, Patient safety, Score, Questionnaire, Self-assessment, CMS, SPADI, DASH, MSQ

## Abstract

**Background:**

Outcome measurement in shoulder surgery is essential to evaluate the patient safety and treatment efficiency. Currently this is jeopardized by the fact that most patient-reported self-assessment instruments are not comparable. Hence, the aim was to develop a reliable self-assessment questionnaire which allows an easy follow-up of patients. The questionnaire also allows the calculation of 3 well established scoring systems, i.e. the Shoulder Pain and Disability Index (SPADI), the Constant-Murley Score (CMS), and the Disabilities of the Arm, Shoulder and Hand (DASH) Score. The subjective and objective items of these three systems were condensed into a single 30-questions form and validated against the original questionnaires.

**Methods:**

A representative collective of patients of our shoulder clinic was asked to fill in the newly designed self-assessment Munich Shoulder Questionnaire (MSQ). At the same time, the established questionnaires for self-assessment of CONSTANT, SPADI and DASH scores were handed out. The obtained results were compared by linear regression analysis.

**Results:**

Fifty one patients completed all questionnaires. The correlation coefficients of the results were r = 0.91 for the SPADI, r = -0.93 for the DASH and r = 0.94 for the CMS scoring system, respectively.

**Conclusions:**

We developed an instrument which allows a quantitative self-assessment of shoulder function. It provides compatible data sets for the three most popular shoulder function scoring systems by one single, short 30-item. This instrument can be used by shoulder surgeons to effectively monitor the outcome, safety and quality of their treatment and also compare the results to published data in the literature.

## Introduction

Continuous follow-up of patient after conservative treatment or surgery is an essential step to monitor the effectiveness of a therapy, to ensure quality management and to improve the patient safety. However, interpretation of shoulder assessment still remains controversial in practice and in literature [1-8]. Multiple scoring systems were proposed, such as the SPADI, DASH, ASES, SRQ, WOOS, SSRS and CMS, based on either examination by a physician and/or additional standardized queries within a questionnaire [6,9-15]. The validity, reliability and responsiveness for most of these instruments were demonstrated in several studies. Hence, these test-systems are widely accepted for outcome measurement in shoulder surgery [1,7,16-18]. However, routine measurement of outcome in shoulder surgery is substantially jeopardized by three major disadvantages of these instruments: i) physical presence of the patient is mandatory if the objective function (e.g. range of motion, muscle strength) is to be assessed, ii) patient-reporting instruments provide only one certain score, making it difficult to compare the results with the literature, iii) several questionnaires are therefore used in most studies but can overburden the compliance of patients willing to participate.

As a result, performing clinical studies is time consuming, expensive and a logistical challenge, especially when physical presence of the patient is required [3-5,16,19]. In addition, many procedures are performed on outpatients in centers far away from the home of the patients. For this reason, many patients will refuse or are just unable to keep a long-term follow-up, beside other issues, such as accidents during travel, insurance etc. This makes it hard to follow-up patients on a regular base and beside the lack of patient safety, valuable data on the outcome and the quality of treatment gets lost. Furthermore, by this patient dropout, the comparison of different treatment regimens gets biased and makes it hard or impossible to state which form of therapy is superior and provides the highest safety level for the patient.

Standard questionnaire enable to follow patients on a close and regular base with manageable effort for both, the surgeon and the patients. An optimal instrument for outcome measurement of shoulder surgery should meet the following requirements:

i) the instrument should be a self-assessment patient reporting tool, so travelling of patients is not required

ii) the questions must be easily comprehensible

iii)filling in should not take more than 30 minutes

iv) the results should provide calculation of several well established scoring systems simultaneously.

Therefore, the aim of this study was to create a self-assessment patient reporting tool by condensing queries of three of the widest distributed and accepted instruments for shoulder function measurement into a 30-item questionnaire based on subjective and objective parameters of shoulder function. This new instrument was then validated by collecting data of the newly designed Munich Shoulder Questionnaire (MSQ) as well as of three established scoring systems (CONSTANT, SPADI and DASH) simultaneously.

## Material and methods

### Development of the questionnaire

We analyzed each single question in the existing questionnaires for CONSTANT, SPADI and DASH scores for congruency in measurement of specific shoulder function items and condensed them into one single question for each specific item. Moreover, typical functional abilities were depicted as photographs, so patients could easily compare their own functional capabilities to the pictures. Thus, we were able to create a single questionnaire asking 30 simple questions which allow for calculating 3 different scoring systems simultaneously.

### The Munich shoulder questionnaire (MSQ)

The MSQ is a 30-items self-assessment questionnaire (Additional file 1). Its raw score ranges from 0 to 314. For comparability, the raw numbers are divided by 314 giving a percentage ranging from 0 to 100 in which higher scores represent a better function of the shoulder.

The MSQ consists of three parts: the cover sheet, one section for the objective and one section for the subjective assessment. The cover sheet is designed to collect demographic information about the patient and his shoulder. Data are obtained as follows: patient’s name, age, sex, affected side, hand dominance, employment, pain medication, the relevant side for which answers are given and the date of completion.

The next section is designed to calculate the objective function of the shoulder and consists of six items. The first five questions assess the range of motion: flexion, abduction, internal rotation, external rotation and range of the hand. Each question offers results from 0 to 10 points resulting in a total score ranging from a minimum of 0 to a maximum of 50 points (16% of the total MSQ). Question 6 targets at the power of the shoulder in 90° of abduction and 20° of flexion. For this item photographs of a model are given demonstrating the position to use (see MSQ question 6). The patient is asked to fill a bag with items of daily living with a defined weight, such as a 17.6 ounce (500g) coffee pack or a 17.6 fluid ounce (500ml = 500g) milk package. Then, the patient is requested to lift the bag as shown to the horizontal plane and hold it for 5 seconds. This is performed stepwise with increasing weights until the maximal feasible load is reached or the maximum of 424 ounces (12kg) is lifted. Each 17.6 once (500g) weight encounters for 2 points giving a maximum of 24 points for this question (8%). Altogether the objective section accounts for a total score ranging from 0 to 74 points (24%).

The second section asks for subjective function and consists of 24 items. Each question allows for an answer with a range from 0 for a poor to 10 for a perfect function. Six of the items deal with pain (19%), nine cover work and daily activities (29%), six cover sports and recreation activities (19%) and three ask for the social and emotional quality of life (10%). Altogether the subjective section accounts for a total score ranging from 0 to 240 points (76%).

### Study collective

For validation a cohort of 56 consecutive patients was randomly selected from our outpatient clinic at the department of surgery. All patients were seen by the senior author (P.B.) between June 2009 and September 2009. Inclusion criteria were as follows: i) written informed consent to participate in the study, ii) unilateral or bilateral shoulder disorder, iii) ability to read and complete four self-assessment questionnaires, iv) no other severe diseases or injuries affecting the result of the shoulder questionnaires. The study protocol was approved by the ethic committee of the university. All patients were informed in detail about the study in advance. The results of the study had no influence on their diagnostics or therapy.

### Questionnaires and scores

At first, the scores of established scoring systems, such as CONSTANT, SPADI and DASH questionnaires were calculated according to the instruction given by the authors. Then, the raw score of the MSQ was taken and computed into a relative percentage by dividing the absolute result by 314. Next the scores of the SPADI, DASH and CMS were calculated from the data of the MSQ (c = calculated: cSPADI, cDASH and cCMS). All questionnaires scale from 0 to 100 with higher scores representing a better function for the SPADI, CMS and MSQ, whereas the DASH score runs inversely with higher scores representing lower function.

### Statistical analysis

The results were compared by calculating Pearson´s correlation coefficient within a linear regression analysis. A p-value <0.05 determined significance. Statistics were calculated using commercially available programs (SigmaStat 3.1, SigmaPlot 8.02, Systat Software GmbH, Erkrath, Germany).

## Results

### Patients and demographic data

From the initial 56 patients five were excluded because of an incomplete questionnaire. 51 patients filled in all questionnaires correctly and were enrolled in the study. 36 patients were male (71%) and 15 female (29%) with a mean age of 51 years (range 20-80 years). 12 patients were twenty to thirty-nine, 26 patients were forty to fifty nine and 13 patients were at least sixty years old. At the day of assessment 33 patients worked as paid form, 3 as housework, 14 were retired and 1 patient was unemployed because of complaints in his shoulder. 41 patients were right handed and 10 were left handed, with 34 patients (67%) having affected the dominant side. The diagnoses of the patients represent the full spectrum of shoulder diseases (Table 1).

**Table 1:** Diagnoses of the 51 patients

**Diagnose**	**Patients**
Dislocation of the AC-joint	4
Osteoarthritis of the shoulder	4
Dislocation of the Shoulder	4
Shoulder pain of unknown origin	2
Rotator cuff tear	3
Impingement	14
Humeral bone cyst	1
Contusion of the shoulder	1
Fracture of the clavicle	1
Fracture of the scapula	1
Fracture of the humerus	13
Damage of the brachial plexus	1
Biceps tendon tear	1
Shoulder arthroplasty	1
**Total number of patients**	**51**

### Questionnaires and scores

**Figure 1:**
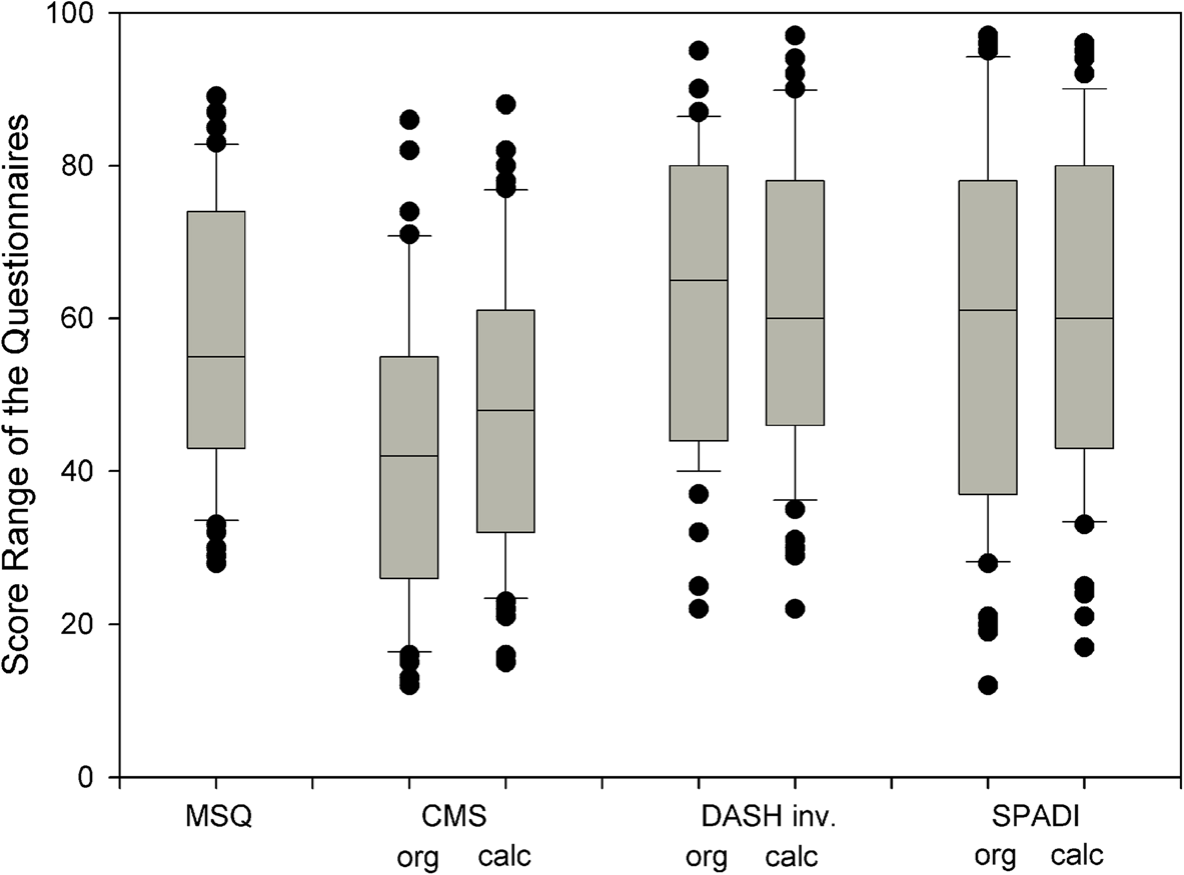
**Box-plots with median, interquartile range and outliers of the results of different shoulder questionnaires.** Original and calculated scores correspond closely. For better comparability the DASH scale has been inverted.

Within the complete collective, the absolute MSQ score was 182 ± 57 points (range 88-310) equivalent to 58 ± 18 [%] (range 28-89) (Figure 1 and Table 2). The original SPADI score accounted for a mean of 60 ± 24 points (range 12-97) and the cSPADI calculated from the MSQ data accounted for a mean of 61 ± 21 points (range 17-96). The mean score of the original CMS was 42 ± 20 points (range 12-86) and the cCMS was 48 ± 19 points (range 15-88). The original DASH score accounted for a mean of 38 ± 19 points (range 5-78) and the calculated cDASH for 39 ± 19 points (3-78 points) (Figure 1 and Table 2).

**Table 2:** Results of the MSQ compared to the established CONSTANT, SPADI and DASH scoring systems

	**Mean**	**SD**	**Max**	**Min**	**Range**
**MSQ**	57.9	17.5	89	28	61
**SPADI org**	59.5	23.7	97	12	85
**SPADI calc**	60.8	21.2	96	17	79
**CMS org**	42.2	19.8	86	12	74
**CMS calc**	47.7	19.3	88	15	73
DASH org inv *	62.2	19.0	95	22	73
DASH calc inv *	61.5	19.2	97	22	75
**DASH org**	37.8	19.0	78	5	73
**DASH calc**	38.5	19.2	78	3	75

* DASH scale inverted for better comparability.

**Figure 2:**
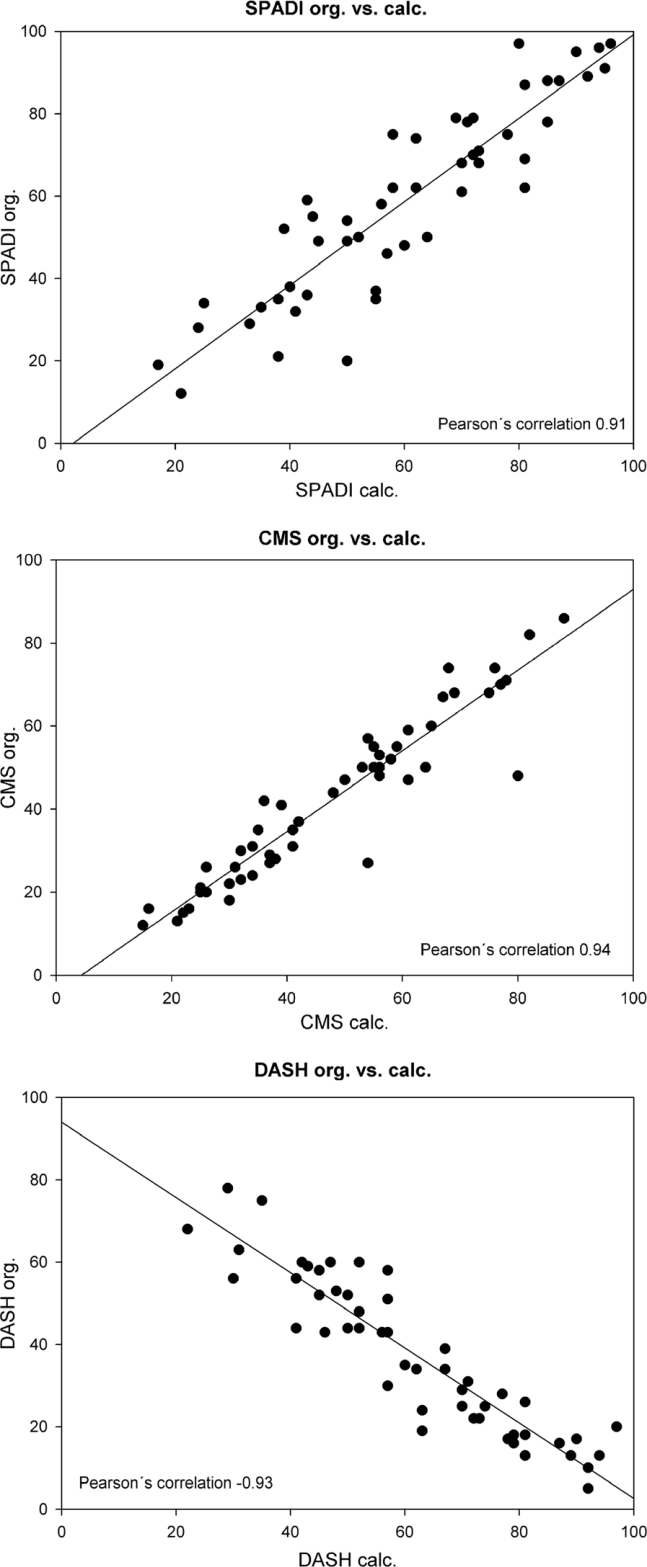
**a - c: Correlation between original and calculated scores as derived from the MSQ.** Pearson´s correlation coefficient for a: the SPADI was 0.91 (p < 0,05), b: the CMS 0.94 (p < 0,05) and c: the DASH -0.93 (p < 0,05).

Comparing the original SPADI and the results taken from the MSQ, Pearson’s correlation coefficient was 0.91 (p < 0.05) (Figure 2). Similar results were obtained for the CMS (r = 0.94 (p < 0.005)). In addition, the scores from the original DASH questionnaire corresponded very well to the cDASH values (r = -0.93, p < 0.05). In this case the r is negative, since the DASH score has an inverse scale (Figure 2).

## Discussion

This study presents an effective instrument for patient-reported outcome measurements in shoulder surgery in order to enable a close and detailed follow-up of patients. It also provides quantitative read outs of 3 of the most widely distributed scoring systems. We believe that this questionnaire is an extremely useful tool for patient monitoring and evaluation of therapy regimes in shoulder surgery and thereby is able to improve the treatment and safety of the patients. Furthermore, it is a self-estimating system and requires no physical presence of the patient and is easy to use, as it supports the patient with exemplary photographs, and consists of only 30 questions that usually requires no longer than 30 minutes to fill in.

In the literature it is widely accepted that shoulder surgery and patient safety would benefit from further outcome studies regarding various therapeutic concepts. However, these studies are jeopardized by several facts: Specialized shoulder surgery is performed in centers. This is why patients have to travel long distances and many procedures are performed on outpatients. Moreover, the comparison of studies is difficult since different scoring systems are in use.

### Features of the questionnaire

#### Requirement No 1: test system should be patient-reporting

The MSQ was designed as a self-assessment instrument offering several advantages compared to a questionnaire requiring physical presence of a physician. First of all it does not require face-to-face contact making it less time consuming for both, the physician and the patient which reduces the drop-out-rate [3,4,12]. Moreover, self-assessment questionnaires require a minimal setup and can be completed by mail making them inexpensive [12]. Furthermore, this eliminates the well-recognized examiner observation or selection bias of physicians rating the patients they treated before much better than other physicians or patients themselves [19]. Finally multiple studies have clearly demonstrated that the evaluation by self-assessment questionnaires is highly reliable, valid and responsive [1,12,20].

#### Requirement No 2: the questions must be comprehensible

To ensure a high response rate the questions were made as simple as possible. The patients were presented photographs of a model demonstrating various positions of the arm, of which the patients could choose their own abilities. This is why the results revealed a significant correlation to well established scoring systems, especially to the Constant score. This concept is in line with other authors, who also developed self-assessment questionnaires [12,19,21]. Furthermore, Boehm et al. also could demonstrate that the CMS can reliably and valid be obtained with a photograph based self-assessment questionnaire [19].

#### Requirement No 3: answering should not expand 30 minutes

Our questionnaire comprises of 30 questions, so the patient has on average one minute for each question, which provides enough time for self-evaluation. Nevertheless, since we condensed several scoring systems into these 30 items, outcome studies using the MSQ are highly effective and a substantial amount of information is created within a short period of time.

#### Requirement No 4: read outs should either stand alone or match with well-established scoring systems for comparability to previous and future studies

The development of the MSQ was based in large parts on three existing and well established scoring systems: the DASH, SPADI and CMS. Generation of items by combining questions of different instruments and thereby reducing their number has already been described by others [22]. Summarizing various items is possible as different or even the same questionnaires often have a considerable overlap or one item is just the more specific form of another [6]. We are convinced to increase the response rate by creating a 30-item questionnaire and thereby reducing the number of applied instruments to a single one. This is especially important for the quality of studies on large collectives and a long follow-up or on treatments performed on outpatients that struggle with high dropout rates.

Despite reduction of the items, the MSQ is able to assess different aspects of shoulder function due to the combination of questionnaires with different focuses. While the CMS mainly evaluates the objective function, and the SPADI and DASH solely address the subjective function of the shoulder, the MSQ captures both [16]. Objective and subjective function, however, have recently been controversially discussed as the objective function of a shoulder alone correlates poorly with the outcome and the quality of life experienced by the patients [16,23,24].

Since examinations made by a physician are laborious and expensive, most of the questionnaires solely focus on subjective assessment. Certainly, the objective function alone does not necessarily reflect the outcome of a therapy but still provides important information when evaluating the effectiveness of a therapy [16]. Especially for comparison of conservative and surgical therapies it is important to gather information on the range of motion and on the power of the affected arm irrespectively from the final rating. This enables the physician to differentiate in which modality a certain therapy offers benefits and helps to determine the specific factors leading to success or failure. By this, the surgeon is also able to review the quality of his work and thus also improve the quality and safety of his treatment.

The availability of a comprehensive scoring system like the MSQ which includes both aspects enhances the ability to evaluate and counsel a patient accurately regarding the effectiveness of an intervention. This is confirmed by other authors who recommend combining subjective rating instruments like the SPADI or DASH score with a questionnaire assessing the objective function or a physical examination [1,12,16]. For this reason, both aspects were integrated in the MSQ. Nevertheless, it is still possible to rate the objective (CMS) and subjective function separately (SPADI and DASH).

### Evaluation and study collective

Evaluation was performed in a collective of 51 patients which was comparable to previous studies concerning the age, gender and diagnoses [11-13,18,19].

Comparison of the original and the calculated scores showed a high degree of correlation for all three. Moreover, comparing the means and standard deviations with respect to the original and calculated scores, almost equal results were found for each questionnaire. Solely the range of the total MSQ was slightly smaller compared to the range of the CMS, DASH and SPADI. This is probably due to the fact that the CMS generally provides lower scores than the SPADI and DASH, resulting in a smaller range when combining them. However, comparing the ranges of the original and calculated scores, similar results were obtained for all of them. Besides, neither the MSQ nor one of the three questionnaires showed any floor and ceiling effects obstructing measurement of a further change in the shoulder function [25].

### Limitation of the study

As a limitation of the study has to be mentioned that single items were not tested separately and the MSQ was not re-evaluated before and after therapy. Since we used well established scoring systems as a draft, we did not develop completely new items. Hence, we used a data pool of existing items from questionnaires which have already been extensively tested. These items correlated clearly with our scores.

## Conclusion

In summary, the presented self-assessment questionnaire allows a comprehensive and reliable rating of the shoulder function. It is easy to use and offers the opportunity to gather data representing the subjective and objective function without the necessity of physical presence. Besides, is possible to calculate the scores of the well-established SPADI, DASH and CMS out of the questionnaire and compare the results with previous studies. Therefore, the MSQ is an effective tool which allows a close follow-up of large patient collectives, aiming to improve the quality of treatment and the safety of patients with a shoulder injury. The MSQ questionnaire is available for free at our homepage http://www.klinikum.uni-muenchen.de/Chirurgische-Klinik-und-Poliklinik-Innenstadt/de/fachgebiete/unfallchirurgie/schulterambulanz/index.html.

## Competing interests

The authors declare that they have no competing interests.

## Authors’ contributions

FS, VBr, EW, PB defined the research goal and designed the methods. FS, MB and VBo collected the data. MB, VBr and VBo analyzed the data. EW and PB interpreted the result and performed statistical analysis. All authors contributed to writing, read and approved the final manuscript.

## Supplementary Material

Additional file 1 Munich shoulder questionnaire. Click here for file
