# Supplementary material for: The Munich Shoulder Questionnaire (MSQ): development and validation of an effective patient-reported tool for outcome measurement and patient safety in shoulder surgery
Source: Patient Saf Surg. 2012 May 18;6:9. doi: 10.1186/1754-9493-6-9 (PMC3502470; doi:10.1186/1754-9493-6-9)
Supplement: Additional file 1 — Munich shoulder questionnaire. [file 1754-9493-6-9-S1.pdf]

Shoulder Questionnaire

Please answer **each question** with exactly **one cross**!

Range of motion

Date:

Sex: ☐ male ☐ female

Affected shoulder: ☐ right ☐ left ☐ both shoulders

Dominant side: ☐ right handed ☐ left handed

Working ☐ yes, as: ____-

☐ no, if no, because of the shoulder? ☐ yes ☐ no

Pensioner ☐

Housewife ☐

Do you take pain medication because of your affected shoulder?

☐ yes if yes, which drugs: ____-

____-

____-

☐ no

For which shoulder do you complete the questionnaire?

☐ right shoulder ☐ left shoulder

© Department of Shoulder Surgery

Flexion

Are you able to lift your stretched arm up to the depicted positions?

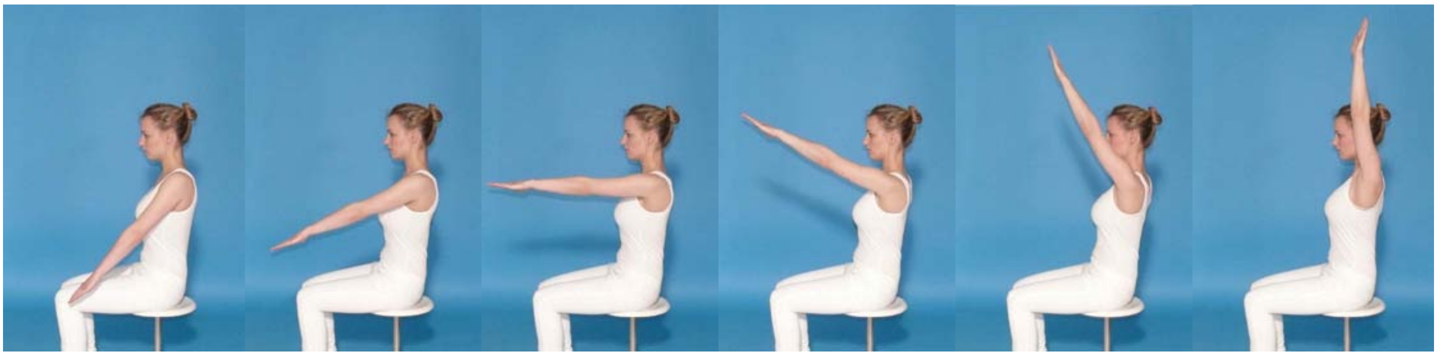

☐ yes	☐ yes	☐ yes	☐ yes	☐ yes	☐ yes
☐ no	☐ no	☐ no	☐ no	☐ no	☐ no

Abduction

Are you able to lift your stretched arm up to the depicted positions?

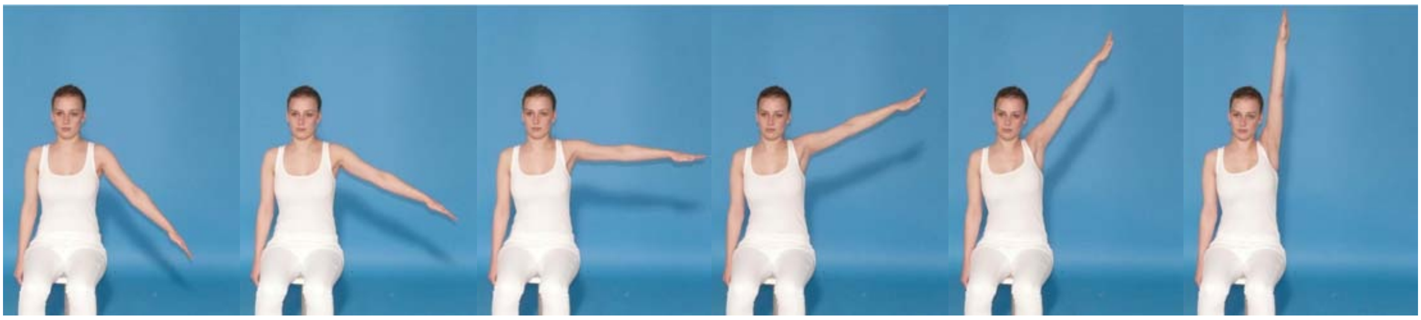

☐ yes	☐ yes	☐ yes	☐ yes	☐ yes	☐ yes
☐ no	☐ no	☐ no	☐ no	☐ no	☐ no

Internal rotation

Up to which height of your back are you able to move the dorsum of your hand?

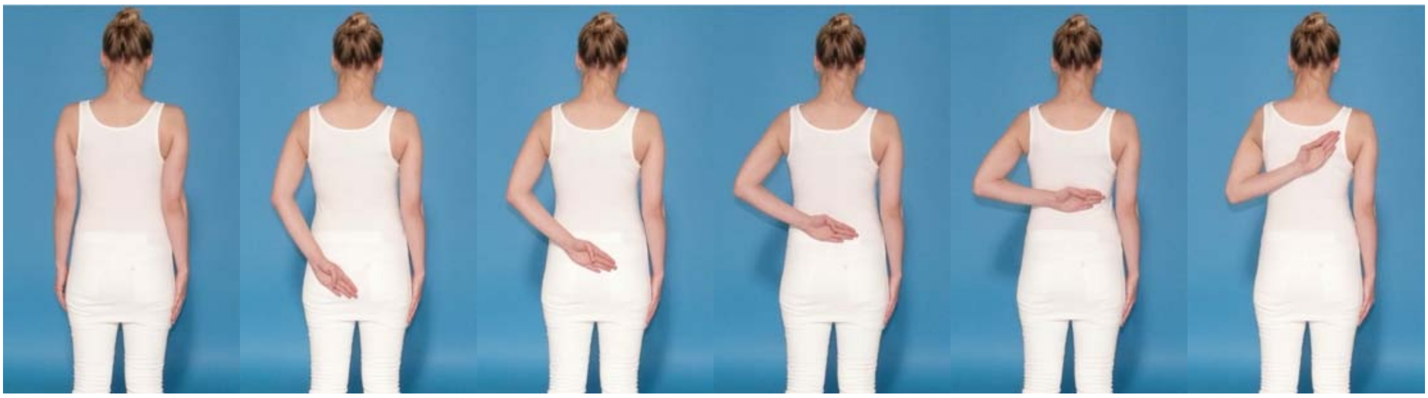

lateral thigh	buttock	tailbone	waistline	upper waist	shoulder blade
☐ yes	☐ yes	☐ yes	☐ yes	☐ yes	☐ yes
☐ no	☐ no	☐ no	☐ no	☐ no	☐ no

© Department of Shoulder Surgery

Positioning

Up to which height are you able to put the palm of your hand?

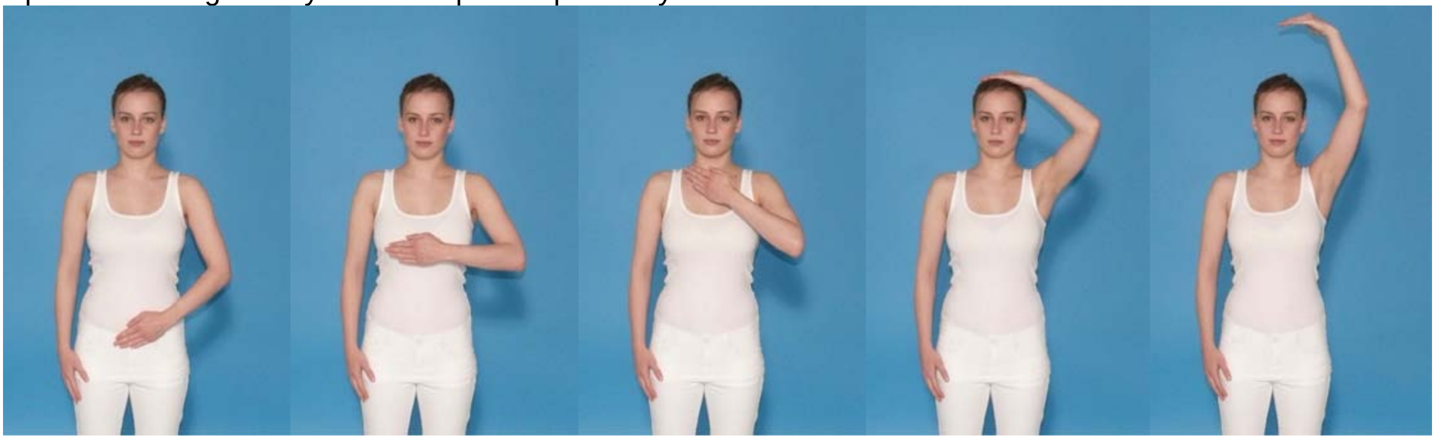

waistline	chest	neck	top of head	above head
☐ yes	☐ yes	☐ yes	☐ yes	☐ yes
☐ no	☐ no	☐ no	☐ no	☐ no

External rotation

Please check exactly one box for each position shown for your affected side:

Are you able to....

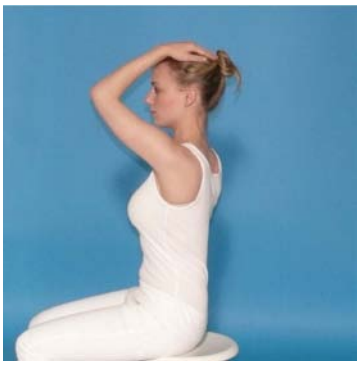

… put your hand on top of the head with the elbow directed forward

☐ yes

☐ no

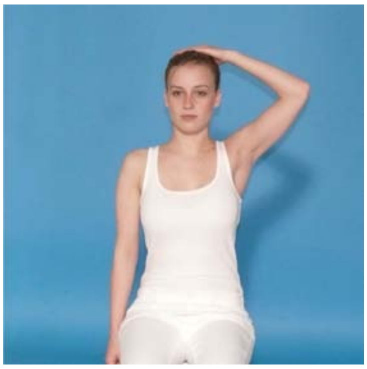

… put your hand on top of the head with the elbow held back

☐ yes

☐ no

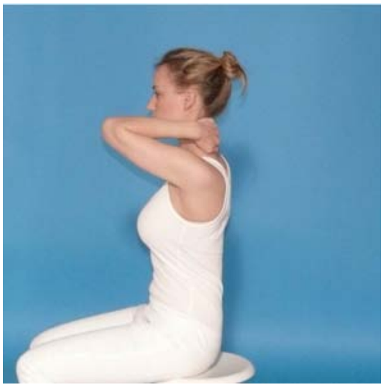

… put your hand in the neck with the elbow directed forward

☐ yes

☐ no

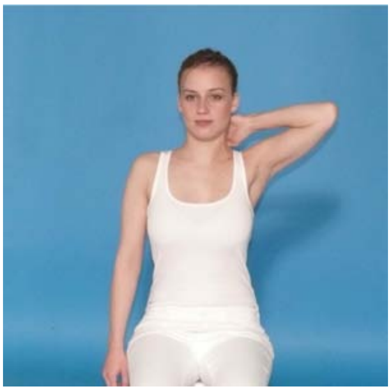

… put your hand in the neck with the elbow held back

☐ yes

☐ no

© Department of Shoulder Surgery

Power of the shoulder:

To measure the power of your affected shoulder you need

- a firm carrier bag
- several objects of known weight

  e.g. coffee pack 17.6 ounce/ (500g),
  or milk tetra-pack 17.6 fluid ounce (500ml=500g)

- Please fill the bag stepwise with 17.6 ounce/ 500g weights, try to lift it according to the depicted position and **hold it for 5 seconds**.

front view:

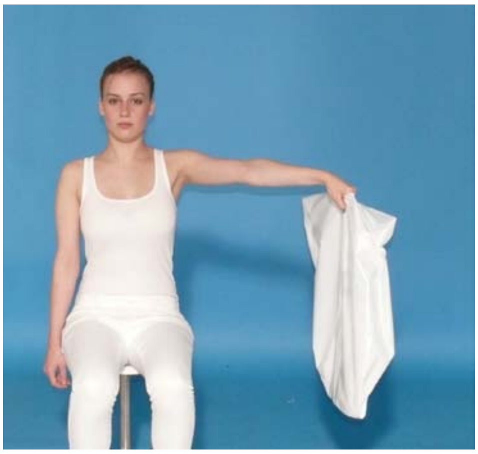

- arm elongated
- back of hand upward
- arm 90° elevation

top view:

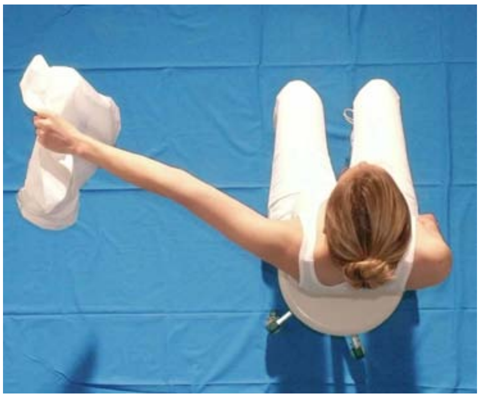

- arm should point about 20° forward

- What is the maximum weight you can lift for five seconds? _____,___ kg

  _____,___ ounce

© Department of Shoulder Surgery

Pain

The following questions ask about pain in your affected shoulder without taking pain killers.

Please check exactly one box of each question that describes your pain best.

How severe was your pain at its worst in the last 14 days?

none ☐ ☐ little ☐ ☐ moderate ☐ ☐ strong ☐ ☐ very strong ☐ ☐ intolerable ☐

How severe is your average shoulder pain when you perform activities of the daily living?

none ☐ ☐ little ☐ ☐ moderate ☐ ☐ strong ☐ ☐ very strong ☐ ☐ intolerable ☐

How severe is your shoulder pain during work/ job?

none ☐ ☐ little ☐ ☐ moderate ☐ ☐ strong ☐ ☐ very strong ☐ ☐ intolerable ☐

How severe is your shoulder pain during recreational activities?

none ☐ ☐ little ☐ ☐ moderate ☐ ☐ strong ☐ ☐ very strong ☐ ☐ intolerable ☐

How severe is your shoulder pain during sleep?

none ☐ ☐ little ☐ ☐ moderate ☐ ☐ strong ☐ ☐ very strong ☐ ☐ intolerable ☐

Do you feel tingling (pins and needles) in your shoulder?

none ☐ ☐ little ☐ ☐ moderate ☐ ☐ strong ☐ ☐ very strong ☐ ☐ intolerable ☐

Please check exactly one box of each question that describes your situation best.

Work & activities of daily living

Are you limited in your work/ profession/ principal activity as a result of your shoulder?

none ☐ ☐ little ☐ ☐ moderate ☐ ☐ strong ☐ ☐ very strong ☐ ☐ intolerable ☐

How much difficulty do you have putting on a pullover?

none ☐ ☐ little ☐ ☐ moderate ☐ ☐ strong ☐ ☐ very strong ☐ ☐ intolerable ☐

© Department of Shoulder Surgery

How much difficulty do you have putting on your pants?

none little moderate strong very strong intolerable

☐ ☐ ☐ ☐ ☐ ☐ ☐ ☐ ☐ ☐ ☐

How much difficulty do you have washing your back?

none little moderate strong very strong intolerable

☐ ☐ ☐ ☐ ☐ ☐ ☐ ☐ ☐ ☐ ☐

How much difficulty do you have carrying an object of approximately 5 kg/11lb sideways (e.g. shopping bag, briefcase)?

none little moderate strong very strong intolerable

☐ ☐ ☐ ☐ ☐ ☐ ☐ ☐ ☐ ☐ ☐

How much difficulty do you have washing or combing your hair?

none little moderate strong very strong intolerable

☐ ☐ ☐ ☐ ☐ ☐ ☐ ☐ ☐ ☐ ☐

How much difficulty do you have placing an object on a shelf above your head?

none little moderate strong very strong intolerable

☐ ☐ ☐ ☐ ☐ ☐ ☐ ☐ ☐ ☐ ☐

How much difficulty do you have to push open a heavy door?

none little moderate strong very strong intolerable

☐ ☐ ☐ ☐ ☐ ☐ ☐ ☐ ☐ ☐ ☐

How much difficulty do you have to manage toileting?

none little moderate strong very strong intolerable

☐ ☐ ☐ ☐ ☐ ☐ ☐ ☐ ☐ ☐ ☐

Recreational activities/ sports

How much difficulty do you have doing easy household chores (e.g. cutting fruits, writing, ironing, turn a key)?

none little moderate strong very strong intolerable

☐ ☐ ☐ ☐ ☐ ☐ ☐ ☐ ☐ ☐ ☐

© Department of Shoulder Surgery

How much difficulty do you have doing medium heavy houshold chores (e.g. cleaning basin, scrubbing pots or pans, washing your clothes by hand)?

none | little | moderate | strong | very strong | intolerable

☐ ☐ ☐ ☐ ☐ ☐ ☐ ☐ ☐ ☐ ☐

How much difficulty do you have doing heavy houshold chores (e.g. shovelling snow, moving the lawn)?

none | little | moderate | strong | very strong | intolerable

☐ ☐ ☐ ☐ ☐ ☐ ☐ ☐ ☐ ☐ ☐

How much difficulty do you have throwing a light ball over your head?

none | little | moderate | strong | very strong | intolerable

☐ ☐ ☐ ☐ ☐ ☐ ☐ ☐ ☐ ☐ ☐

How much difficulty do you have to participate in easy sports (e.g. miniature golf, bowling, boccia, frisbee)?

none | little | moderate | strong | very strong | intolerable

☐ ☐ ☐ ☐ ☐ ☐ ☐ ☐ ☐ ☐ ☐

How much difficulty do you have to participate in heavy sports (e.g. swimming, golf, volleyball, tennis)?

none | little | moderate | strong | very strong | intolerable

☐ ☐ ☐ ☐ ☐ ☐ ☐ ☐ ☐ ☐ ☐

Social life

How much difficulty do you have to use public transportation (bus, metro)?

none | little | moderate | strong | very strong | intolerable

☐ ☐ ☐ ☐ ☐ ☐ ☐ ☐ ☐ ☐ ☐

To what extent does your shoulder interfere with your normal social activities with family, friends, neighbours or groups?

none | little | moderate | strong | very strong | intolerable

☐ ☐ ☐ ☐ ☐ ☐ ☐ ☐ ☐ ☐ ☐

Do you feel less capable, less confident or less useful because of your shoulder problem?

never | rarely | sometimes | often | very often | always

☐ ☐ ☐ ☐ ☐ ☐ ☐ ☐ ☐ ☐ ☐

Thank you very much for your efforts!

© Department of Shoulder Surgery
